# Negative Regulation of the Androgen Receptor Gene Through a Primate-Specific Androgen Response Element Present in the 5′ UTR

**DOI:** 10.1007/s12672-014-0185-y

**Published:** 2014-06-04

**Authors:** Colin W. Hay, Kate Watt, Irene Hunter, Derek N. Lavery, Alasdair MacKenzie, Iain J. McEwan

**Affiliations:** School of Medical Sciences, University of Aberdeen, Foresterhill, Aberdeen, AB25 2ZD UK

## Abstract

The androgen receptor (AR) is a widely expressed ligand-activated transcription factor which mediates androgen signalling by binding to androgen response elements (AREs) in normal tissue and prostate cancer (PCa). Within tumours, the amount of AR plays a crucial role in determining cell growth, resistance to therapy and progression to fatal castrate recurrent PCa in which prostate cells appear to become independent of androgenic steroids. Despite the pivotal role of the AR in male development and fertility and all stages of PCa development, the mechanisms governing AR expression remain poorly understood. In this work, we describe an active nonconsensus androgen response element (ARE) in the 5′ UTR of the human AR gene. The ARE represses transcription upon binding of activated AR, and this downregulation is relieved by disruption of the regulatory element through mutation. Also, multiple species comparison of the genomic region reveals that this ARE is specific to primates, leading to the conclusion that care must be exercised when elucidating the operation of the human AR in PCa based upon rodent promoter studies.

**Electronic supplementary material:**

The online version of this article (doi:10.1007/s12672-014-0185-y) contains supplementary material, which is available to authorized users.

## Introduction

In male humans, androgens induce development of the prostate gland during the second and third trimester from the endodermally derived urogenital sinus through epithelial-mesenchymal interactions that lead to epithelial proliferation, invasion, and bud formation (reviewed in Prins and Putz [1]). Circulating testosterone is reduced to the more potent dihydrotestosterone (DHT) that binds to the androgen receptor (AR) causing transformational change and activation. Thereafter, the androgen receptor choreographs differentiation and growth of normal prostate epithelial cells through the coordination of multiple signalling pathways and developmental genes including *sonic hedgehog* (*Ssh*), the *Notch* pathway, *wnt* operating through nuclear β-catenin, *Nkx3.1*, *Hoxb13* and *Sox9*. Unfortunately, the AR signalling axis can also actuate and stimulate carcinogenesis of the prostate, and prostate cancer (PCa) is now the second most common cancer in men in Western nations, with comparable figures rising in Asian countries [2].

Although multiple mechanisms contribute to the lethal progression from benign prostatic hyperplasia to metastatic cancer, AR-mediated cell signalling continues to govern cell growth and survival [3–5] with many of the androgen-induced developmental programmes being reactivated in an aberrant manner during malignant prostatic initiation and growth [6], e.g. overexpression of β-catenin [7] and Sox9 [8]. Consequently, androgen ablation therapy through inhibition of AR function with antagonists or abatement of testicular or intratumoural androgen synthesis form the basis of treatment [3, 9]. Tumours invariably advance to a state referred to as castrate recurrent PCa (CRPC) [10] where they become independent of circulating androgen with a concomitant bleak prognosis [11]. Androgen signalling plays a pivotal role in PCa and increased AR expression is a common feature of both primary tumours and metastases, with a high AR profile in the latter correlating to larger tumour size [12]. The majority of CRPC tumours overexpress AR [13–15], and the AR gene is amplified in approximately a third of cases [16]. The resulting elevated levels of receptor allow cancer cells to be stimulated by low concentrations of androgen [17], which continue to be synthesised in CRPC [18], and afford protection from high-dose antiandrogen therapy, e.g. treatment with bicalutimide [19].

The androgen receptor exerts its influence by acting as an androgen-activated transcription factor which binds to androgen response elements (AREs) [3] where hierarchical complexes of cofactors and other transcription factors govern the transcriptional response [20]. The binding of AR to AREs can elicit stimulation or repression depending upon the relative intracellular concentrations of coactivators and corepressors, and the specific sequence of the ARE and surrounding chromatin architecture [21]. Within the PCa genome, the overwhelming majority of delineated AR binding sites (86 to 95 %) are located outside the promoters of AR responsive genes necessitating chromatin looping [22]. The human single copy AR gene is located at Xq11.2-q12 and possesses a promoter lacking TATA and CAAT boxes, and several regulatory elements have been mapped (see [23] for review). The 4.3-kb AR transcript has an unusually long 5′ untranslated region (5′ UTR) of 1.1 kb. It has been recognised for some time that the AR gene is subject to auto-downregulation in many androgen target tissues, including the human PCa cell line LNCaP, with the androgen-mediated response occurring at the level of reduced messenger RNA (mRNA) transcription [24–29].

Despite the fundamental importance of AR levels in all stages of PCa progression, the *cis*-acting regulatory sequences involved in androgen-mediated downregulation of AR mRNA remain poorly understood with only one site in the second intron described in detail to date [30]. Conversely, four AREs located within exons 4 and 5 have been identified and shown to mediate AR-dependent upregulation of receptor mRNA (reviewed in [31]). In this report, we describe an active nonconsensus androgen response element in the 5′ UTR of the human AR gene that binds AR and elicits repression of AR transcription. Disruption of the ARE by mutation relieves this negative regulation of the AR gene in PCa cell lines expressing AR but not in DU145 which does not express endogenous AR. Therefore, the potential detrimental effects of androgen deprivation therapy (ADT) on PCa tumour development through increased AR transcription should be borne in mind. Lastly, comparison of the genomic region in multiple species reveals that this ARE is specific to primates, necessitating caution in extrapolating findings from rodent promoter studies to the etiology and treatment of prostate cancer.

## Materials and Methods

### Cell Culture

Human prostate carcinoma cell lines LNCaP and VCaP were obtained from the European Collection of Cell Cultures, and DU145 was from the American Type Culture Collection. VCaP and DU145 were grown in DMEM while LNCaP were maintained in RPMI containing 1 mM Na pyruvate and 10 mM HEPES. All media were supplemented with either 10 % foetal bovine serum or 10 % charcoal-stripped foetal bovine serum (both from PAA) and maintained at 37 °C without antibiotics in a humidified atmosphere containing 95 % air and 5 % CO_2_.

### RT-PCR

LNCaP or VCaP cells were grown in medium containing charcoal-stripped serum to approximately 70 % confluence and then cultured for a further 24 h in complete medium containing either 10 nM DHT or ethanol vehicle. Extraction of RNA and RT-PCR were carried out as described earlier [32]. Semiquantitative PCR for hAR and GAPDH was performed under conditions of linear amplification (30 and 26 cycles of amplification for hAR and hGAPDH, respectively) using the primers: hAR-Forward, 5′-TATCCCAGTCCCACTTGTGTC-3′; hAR-Reverse, 5′-CTTGTGCATGCGGTACTCATTG-3′; GAPDH-Forward, 5′-CGGAGTCAACGGATTTGGTCG-3′ and GAPDH-Reverse, 5′-CAATGCCAGCCCCAGCGTCA-3′. The GAPDH primers were specific for mRNA and did not amplify pseudogenes. The resulting DNA products were resolved by 2.0 % agarose gel electrophoresis in TAE buffer (40 mM Tris-acetate, 1 mM EDTA pH 8.3) and visualised by ethidium staining. Integration analysis of gels made use of the Image J software package using exposures that contained no pixel saturation.

### Plasmids and Site-Directed Mutagenesis

The luciferase reporter plasmid phAR1.6Luc, in which luciferase expression is driven by the promoter and 5′ UTR of the human androgen receptor gene, was created using the promoterless firefly luciferase vector pGL4.17 (Promega). The region of the human AR gene spanning between −741 and +842 was amplified by PCR using human male placental genomic DNA template (Cambio), the oligonucleotides 5′-GTTTACAGAGCTCTGGACAAAATT-3′ and 5′-TTCAAAAGATGCCCAGATCTTAAAA-3′, and Pfu Turbo ultrahigh fidelity DNA polymerase from Stratagene. The cloned hAR genomic DNA was digested with SacI and BglII (sites underlined in the PCR primers) and ligated into the SacI and BglII sites in the vector.

Both half sites within a potential androgen response element (ARE) in the hAR 5′ UTR of phAR1.6Luc were mutated using the QuikChange II Mutagenesis kit (Agilent Technologies) according to the manufacturer’s protocol. Mutagenesis was performed in two sequential rounds to create phAR1.6Luc-AREm using the following oligonucleotides with their reverse complements (mutated bases shown in bold font): AREm1, 5′-GGTTAGGCTGCACGCGGAGA**CTGT**CCTCTGTTTTCCCCCAC-3′ followed by AREm2 5′-CACGCGGAGACTGTCCTC**GCAG**TTCCCCCACTCTCTCTCC-3′. The integrity of all constructs was confirmed by DNA sequencing.

### Transfection and Luciferase Reporter Gene Assays

Twenty-four-well plates were seeded with LNCaP and VCaP cells at a density of 5 × 10^4^ cells/cm^2^, while DU145 cells were seeded at a density of 1.2 × 10^4^ cells/cm^2^. The cells were cultured in complete medium for 24 h, then transfected with 440 ng/well of either firefly luciferase reporter plasmid alone or cotransfected with pSVARo human androgen receptor expression plasmid (2:1 ratio) using jetPEI polyethylenimine transfection reagent (Polyplus Transfection) according to the manufacturer’s protocol. After 24 h, the medium was replaced and the cells were cultured for a further 48 h.

Plasmid transfection was performed in quadruplicate and luciferase activity was measured in duplicate by using a GloMax 96 Microplate luminometer (Promega) and normalised for protein concentration as previously described [33].

### Preparation of Nuclear Extracts and Purified Human Androgen Receptor

Nuclear extracts were prepared from LNCaP cells in the presence of protease inhibitors (complete protease inhibitor cocktail from Roche plus 1.0 mM PMSF) and protein phosphatase inhibitors (5 mM β-glycerophosphate and 100-μM activated Na_3_VO_4_) using the method of Dignam et al. [34].

GST-tagged proteins encompassing the hAR N-terminal domain (NTD) plus DNA-binding domain (DBD) or DBD alone (amino acids 1–645 and 529–645, respectively, with numbering based on hAR with NTD repeats of 21 glutamines and 16 glycines) were expressed and purified as described previously; the GST tags were removed by digestion with thrombin (GE Health Care) [35]. The protein concentration of nuclear extracts and hAR fragments were determined using the Bio-Rad DC Protein Assay (Bio-Rad) with BSA as a standard.

### Electrophoretic Mobility Shift Assays

Either 10 μg LNCaP cell nuclear extract or 200 nM recombinant hAR-DBD or hAR-NTD-DBD proteins were incubated with 20 fmol biotin 3′ end-labelled double-stranded DNA oligonucleotides using previously described conditions. The forward sequences of the oligonucleotides were as follows: ARE, 5′-ACGCGGAGAGAACCCTCTGTTTTCCCCCAC-3′; AREm, 5′-ACGCGGAGACTGTCCTCGCAGTTCCCCCAC-3′; and PSA-ARE-III, 5′-ACTCTGGAGGAACATATTGTATCGATTGTC-3′. Unlabelled versions of these oligonucleotides, along with a random oligonucleotide (RO), 5′-CGAGCACCCTTCACCCTCCAGGCTTAACGG-3′, containing no regulatory elements were used for competition assays in which they were added 15 min prior to the labelled probe. Similarly, AR441 antibody against human androgen receptor (sc-7305, SantaCruz Biotechnology) was added 15 min prior to the addition of labelled probe for supershift assays.

The resulting DNA:protein products were resolved in cooled 6 % nondenaturing polyacrylamide gels run in 0.5× TBE buffer, pH 8.3 (45 mM Tris-borate, 1 mM EDTA) and detected using Pierce LightShift Chemiluminescent reagents (Thermo Scientific) according to the manufacturer’s protocol. Figures were compiled using autorads of electrophoretic mobility shift assay (EMSA) gels with the order of lanes within some gels being altered to aid clarity and facilitate comparisons. Digital integration of the DNA:protein complexes was carried out using a Vilber Loumat Fusion SL cooled CCD sensor with care being taken to ensure that no pixel saturation occurred.

### Chromatin Immunoprecipitation Assay

A detailed account of the chromatin immunoprecipitation (ChIP) methodology is presented in Electronic Supplementary Material. In brief, LNCaP cells were transfected with either phAR1.6Luc or phAR1.6Luc-AREm and later treated with either 10 nM DHT or vehicle for 4 h. Cells were fixed in 1 % formaldehyde for 10 min at 37 °C, and nuclei were prepared. Chromatin and plasmid were digested with 400 units each PvuII (NEB) and NheI (Roche) for 15 min at 37 °C; followed by lysis and the removal of insoluble debris by centrifugation. The supernatant was diluted in ChIP buffer and precleared using Protein G and Protein A Dynabeads (Life Technologies). Samples of cleared lysates were retained as input (IP), and the remainder was incubated with either anti-hAR antibody (PG21, 06-680 Millipore) or IgG. Immunocomplexes were collected by magnetisation, washed twice each with low salt, high salt and LiCl and TE buffers, followed by elution. DNA-protein cross-links were reversed with NaCl and DNA purified. Isolated DNA was quantified by semiquantitative log phase PCR and resolved by agarose gel electrophoresis in TAE buffer. The forward (F) and reverse (R) primers were as follows: ARE-F, 5′-CATTGCAAAGAAGGCTCTTAGG-3′; Cont-F, 5′-CCCGAGTTTGCAGAGAGGTA-3′; Gen-R, 5′-GGACAAGATCTGCCCTGCTA-3′; Vect-R, 5′-TCTTCCATGGTGGCTTTACC-3′; PSA-ARE-III-F, 5′-GGTGAGAAACCTGAGATTAGGAATC-3′ and PSA-ARE-III-R, 5′-GTGTGTCTTCTGAGCAAAGACAGC-3′.

### Statistical Analysis

The statistical significance of differences in data sets of DNA:protein complex formation in EMSA experiments was determined using two-way ANOVA, and paired *t*-test analysis of variance was employed for all other comparisons between complementary data.

## Results

### A Primate-Specific Androgen Response Element Is Present in Human AR Gene 5′ UTR

**Fig. 1:**
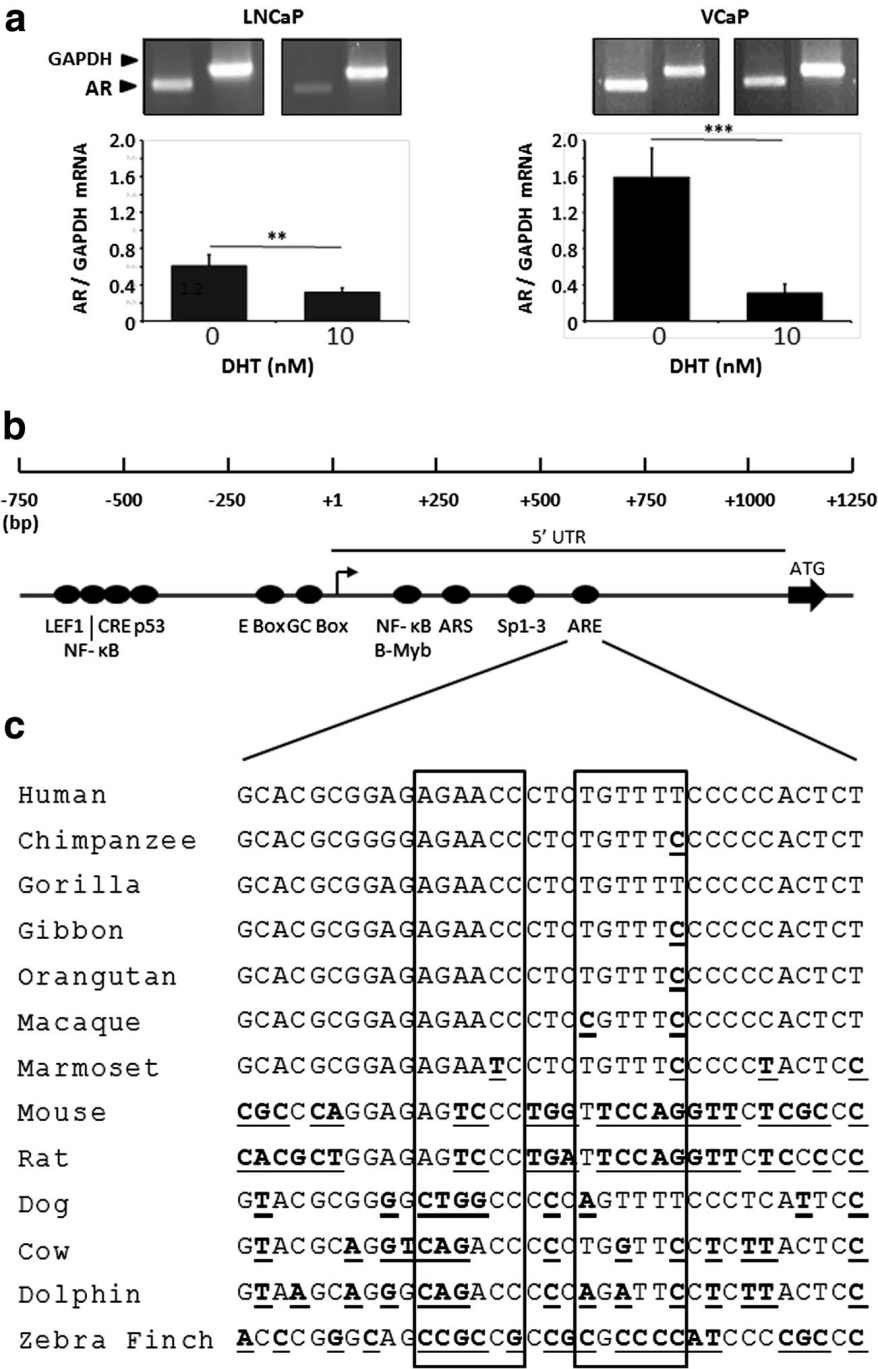
Non-consensus *ARE* in hAR 5′ UTR. **a** Semiquantitative RT-PCR analysis of endogenous AR gene expression in LNCaP or VCaP cells following treatment with either 10 nM DHT or ethanol vehicle. The data represent the means ± SD of at least three independent experiments and statistical significances are the following: **p < 0.01; ***p < 0.001. **b** Diagrammatic representation of the human AR gene proximal promoter and 5′ UTR showing the principal regulatory elements and putative nonconsensus *ARE*. Bent arrow indicates the transcriptional start site (+1) and ATG with *solid arrow* shows the start of translation. **c** Alignments of the putative *ARE* region in AR gene 5′ UTRs of the indicated species with the two half sites demarked by *boxes*. Differences from the human sequence are indicated by *bold*, *underlined* font

Autoregulation of the AR gene by androgens is likely to play an important role during development and in conditions where circulating androgen levels have been reduced. To confirm earlier observations that androgens downregulate AR gene expression, LNCaP and VCaP cells were treated with 10 nM DHT followed by isolation of the RNA. Figure 1a shows that, relative to the transcript of the housekeeping enzyme GAPDH, transcription of the hAR gene in the absence of androgen was markedly higher in VCaP than in LNCaP (*p* < 0.01). Conversely, treatment with androgen reduced AR mRNA in VCaP to a much greater degree than in LNCaP with values of 80 and 48 %, respectively.

Bioinformatic analysis of publically available DNA sequences was used to identify possible AREs in the promoter and adjacent proximal and distal sequences of the human androgen receptor (hAR) gene. Only a previously described suggested nonconsensus ARE (AGAACCctcTGTTTT) at position 611 bp in the 5′ UTR of exon 1 [36] was revealed (Fig. 1b). The putative ARE contains two half sites which are separated by three nucleotides and form a partial palindromic repeat; analogous to a canonical class 1 ARE. Comparison of the equivalent region of the AR gene 5′ UTR in 13 species using multiple alignments (Fig. 1c) showed that this sequence is present only in primates. Gorilla, which diverged from humans 8.6 million years ago [37], has a perfect homology with human, and over the span of 42.2 million years from the divergence of humans and marmoset, the most distant primate examined, the majority of sequences show only a single nucleotide substitution. This is in marked contrast to all of the nonprimate species which possess low levels of homology with human, and no equivalent sequence was found in fish species.

### Androgen Receptor Binds to the Putative ARE

**Fig. 2:**
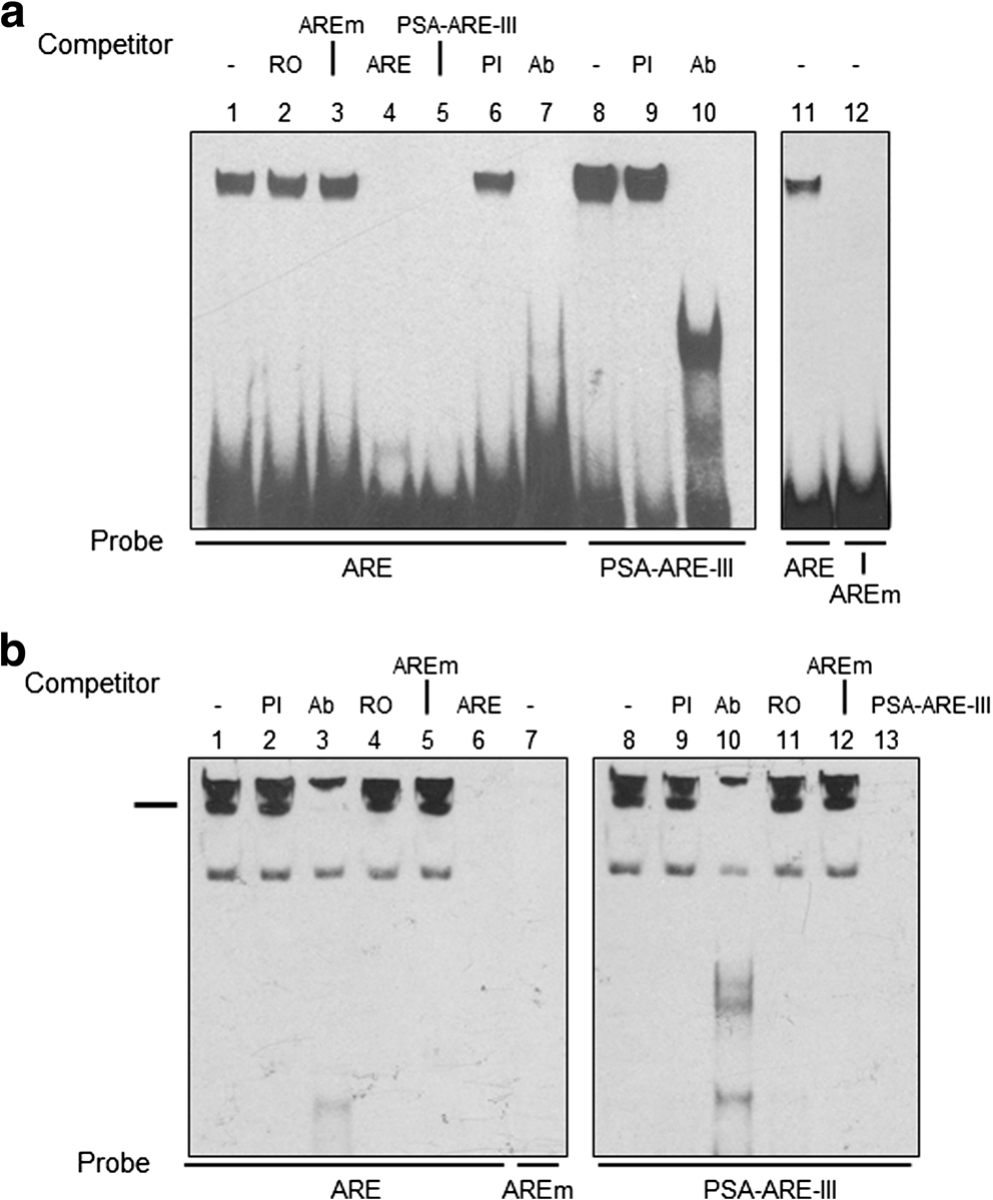
Androgen receptor binds to the 5′ UTR *ARE*. Purified hAR protein or nuclear extract from LNCaP cells were incubated with the labelled oligonucleotide probes indicated below each gel and the products resolved by electrophoretic mobility shift analysis. Competing unlabelled oligonucleotides (100-fold molar excess) or immune sera added prior to addition of probe are shown above the gels. EMSAs are representative of at least three independent experiments. **a** Purified hAR protein encoding the NTD and DBD (residues 1–645) was incubated with labelled probe. Additions were the following: *RO* a random oligonucleotide; *PI* preimmune serum and Ab, anti-hAR-NTD antibody. **b** Nuclear extract from AR-expressing LNCaP cells was incubated with labelled probe, and the complex absent after incubation with antibody is indicated by the marker. These gels were electrophoresed for an additional 30 min to resolve the high molecular weight complexes

The possibility that hAR binds to the nonconsensus ARE was examined by electrophoretic mobility shift assays (EMSAs). In initial experiments, purified hAR protein encoding the N-terminal domain (NTD) and DNA-binding domain (DBD), i.e. amino acids 1 to 645 was incubated with labelled oligonucleotide probe (ARE) containing the putative 5′ UTR ARE. Electrophoretic resolution of the resulting products showed a single high molecular weight DNA:protein complex near the top of the gel (Fig. 2a, lane 1). In addition, Fig. 2a lanes 1 and 8 show that this DNA:protein complex had very similar characteristics to that created with a labelled oligonucleotide (PSA-ARE-III) encoding the well-characterised, active ARE present in the upstream enhancer of the androgen-regulated PSA gene at position −4,200 bp [38]. Binding of hAR NTD-DBD to oligonucleotide ARE was unaffected by preincubation with an excess of a random oligonucleotide (RO) containing no regulatory elements as determined by TRANSFAC analysis or one in which both half sites of the ARE had been mutated (AREm); however, oligonucleotides ARE and PSA-ARE-III completely prevented DNA:protein complex formation (Fig. 2a, lanes 2 to 5, respectively). Preincubation with preimmune serum had no effect on binding of hAR NTD-DBD to either oligonucleotide ARE or PSA-ARE-III (Fig. 2a, lanes 6 and 9, respectively), whereas anti-hAR antibody AR441, against an epitope between amino acids 299 and 315 in the NTD, effectively blocked binding of hAR to both oligonucleotides (Fig. 2a, lanes 7 and 10, respectively). Incubation of hAR NTD-DBD with labelled oligonucleotide containing the mutated form of the ARE failed to produce DNA:protein complex (Fig. 2a, lanes 11 and 12). Similar results were observed using just the DNA-binding domain of hAR (amino acids 529 to 645) and are shown in Supplemental Fig. 1a.

Incubation of nuclear extract prepared from the AR expressing prostate cancer cell line LNCaP with either ARE or PSA-RE-III oligonucleotides produced several bands with virtually identical electrophoretic motilities, but not with the mutated AREm (Fig. 2b, lanes 1, 8 and 7, respectively). Binding of hAR to ARE was confirmed by addition of anti-hAR antibody AR441 which completely prevented assembly of a high molecular weight DNA:protein complex with both ARE and PSA-ARE-III (Fig. 2b, lanes 3 and 10, respectively), while preimmune serum (PI) had no effect (Fig. 2b, lanes 2 and 9). Competing oligonucleotides lacking ARE sites, i.e. RO and AREm, failed to inhibit DNA:protein complex formation with both ARE and PSA-ARE-III probes (Fig. 2b, lanes 4 and 5 and 11 and 12, respectively) while positive controls using 50- or 100-fold excess of unlabelled self-competitors did (Fig. 2b, lanes 6 and 13).

### Comparative Affinities of 5′ UTR ARE and PSA-ARE-III for hAR

**Fig. 3:**
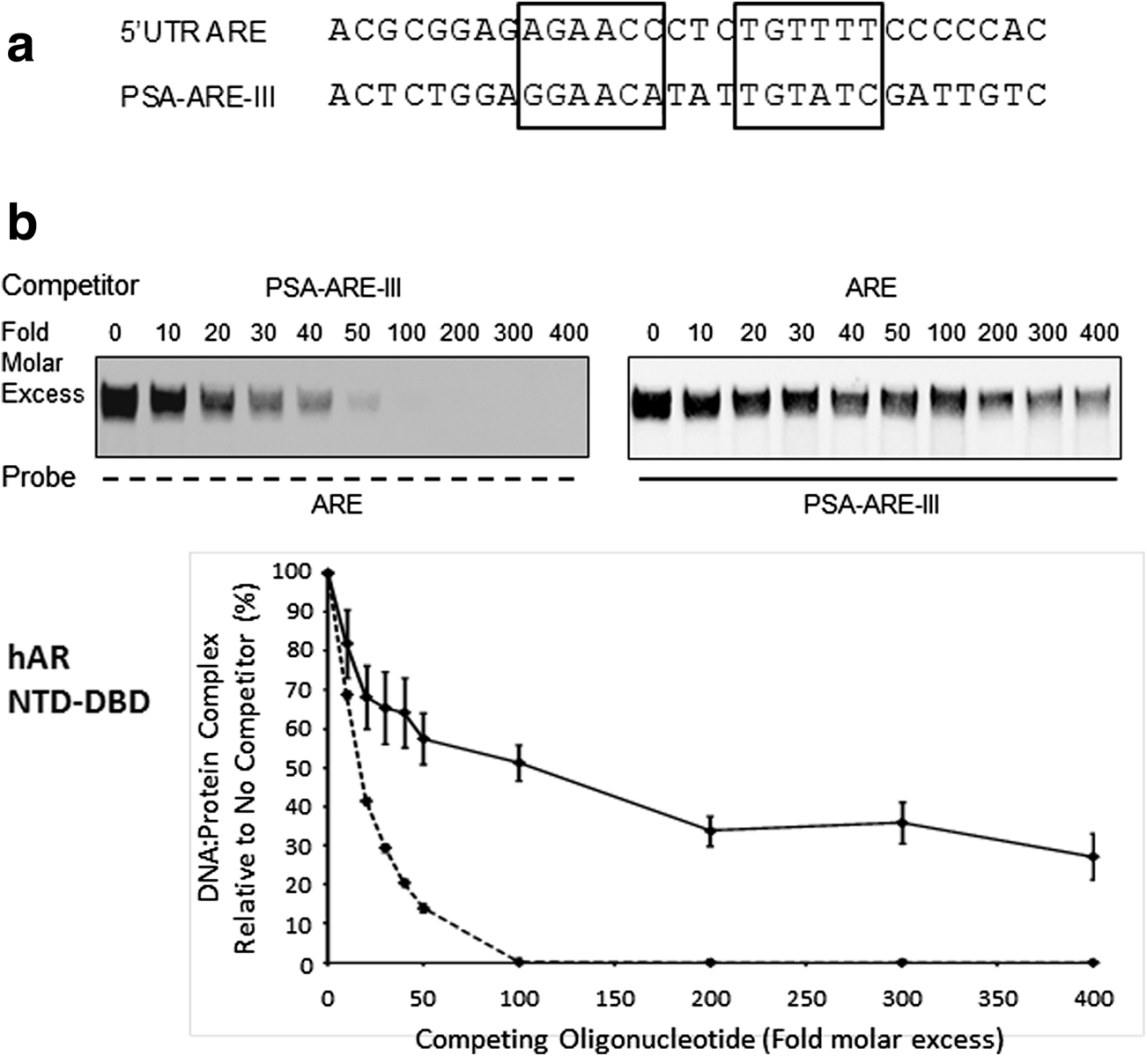
The nonconsensus 5′ UTR *ARE* has lower affinity for hAR than does a consensus ARE. **a** Comparison of the 5′ UTR ARE and *PSA-ARE-III* oligonucleotides used in EMSAs. **b** Human AR NTD-DBD was incubated with either labelled *ARE* probe and competed by preincubation with *PSA-ARE-III* oligonucleotide (*dashed line*) or labelled *PSA-ARE-III* probe and competed with *ARE* oligonucleotide (*solid line*). The molar excess of unlabelled competing oligonucleotide is shown above representative EMSAs. Data presented in the graphs were generated using unsaturated images, and the values are the means of a minimum of three independent experiments ± SD

The EMSA in Fig. 2a suggested differential binding of hAR NTD-DBD to the 5′ UTR ARE in comparison to PSA-ARE-III. This was investigated further by carrying out a series of EMSAs using a constant amount of these labelled probes after preincubation of a fixed amount of hAR protein with a range of excess competing unlabelled oligonucleotide, followed by integration of the digital gel images (Fig. 3). The results showed that competing PSA-ARE-III oligonucleotide prevented hAR NTD-DBD binding to ARE much more effectively than the converse situation (Fig. 3, *p* < 0.001). A similar finding was obtained using the hAR DBD (Supplemental Fig. 1b, *p* < 0.001). Together these results show that hAR binds to the nonconsensus 5′ UTR ARE; however, it does so with lower affinity than to ARE-III in the PSA enhancer.

### The 5′ UTR ARE Downregulates Promoter Activity

**Fig. 4:**
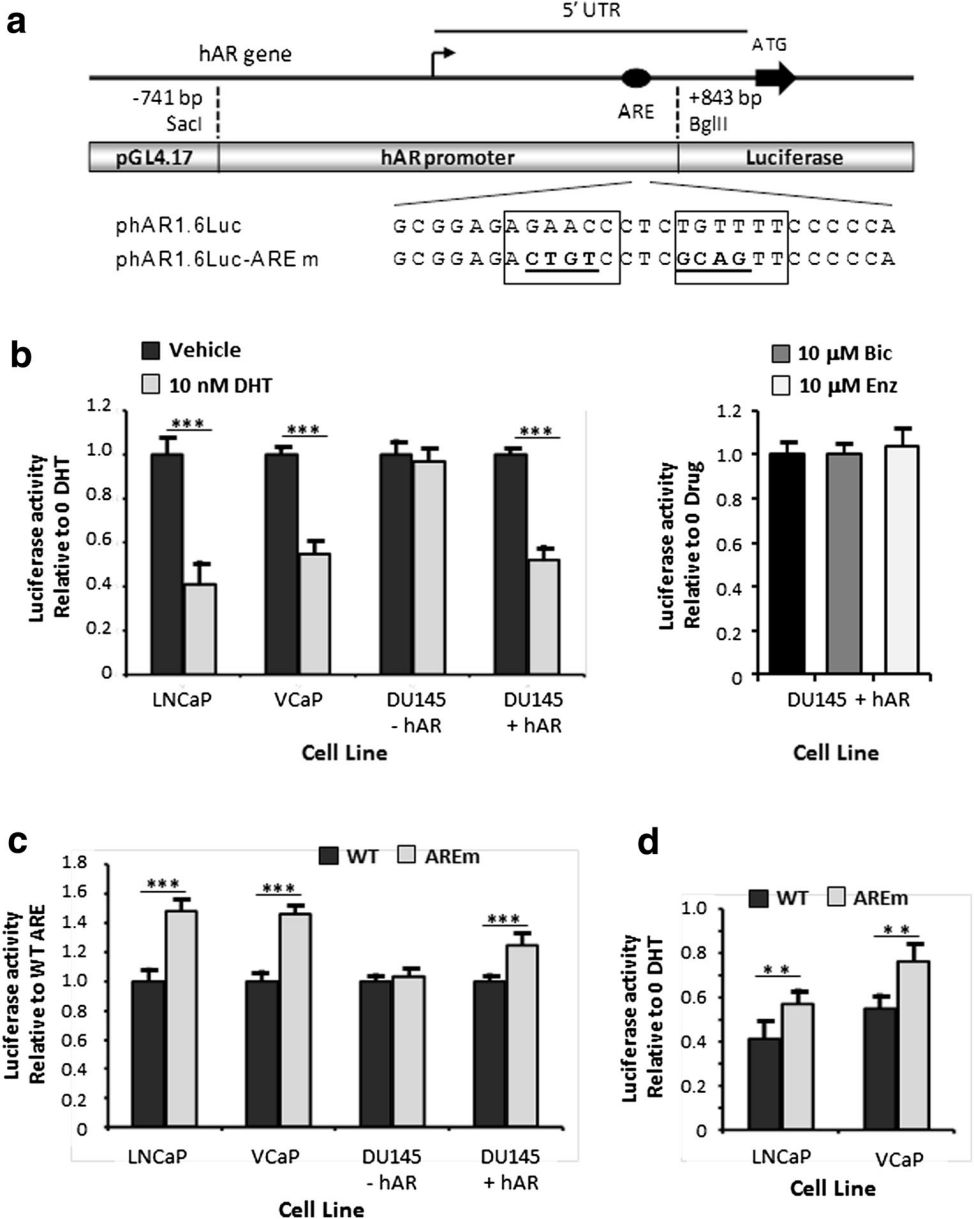
The 5′ UTR *ARE* downregulates promoter transcriptional activity. **a** Schematic representation of the 1.6-kbp section of the hAR promoter and 5′ UTR used to drive luciferase expression in reporter construct phAR1.6Luc. *Bent arrow* indicates the transcriptional start site and mutation of the ARE half sites (*boxed*) are *underlined*. **b** Effect of DHT and antiandrogens on hAR promoter activity in PCa cell lines. *Left panel*: the indicated PCa cell lines were transfected with phAR1.6Luc containing the WT *ARE* and treated with either 10 nM DHT or vehicle. The values show luciferase activity in cells treated with DHT relative to those cultured in vehicle for each given cell line. *Right panel*: luciferase activity for DU145 cells expressing the hAR treated with 10 μM bicalutamide (Bic) or 10 μM enzalutamide (Enz) relative to those cultured in vehicle. Mean ± SD for a representative experiment. **c** The indicated PCa cell lines were transfected with either phAR1.6Luc (WT) or phAR1.6Luc-AREm (AREm) and cultured in complete medium. The values show luciferase activity of the mutated reporter plasmid relative to that encoding the WT ARE for each cell line. **d** The PCa cell lines LNCaP and VCaP were transfected with either phAR1.6Luc (WT) or phAR1.6Luc-AREm (AREm) and treated with 10 nM DHT or vehicle. The values show luciferase activity in cells treated with DHT relative to those cultured in vehicle for each given cell line and plasmid. Luciferase data represent the means ± SEM of at least three independent experiments and the statistical significance of the indicated comparisons are the following: *p < 0.05; **p < 0.01; ***p < 0.001

In order to determine whether the putative ARE had in vivo functional activity, a 1.6-kbp section of the hAR promoter and 5′ UTR (between positions −741 to +842 bp) was cloned into the pGL4.17 promoterless luciferase reporter plasmid to create phAR1.6Luc (Fig. 4a). This region contains the crucial GC box in the TATA-less promoter and the main regulatory elements (see Fig. 1a), thus ensuring that the putative ARE would operate in a normal, physiologically relevant manner. Initial experiments involved studying the response of phAR1.6Luc to androgen in several prostate cancer cell lines by carrying out transient transfection followed by treatment with either 10 nM DHT or vehicle. The results in Fig. 4b (left panel) show that DHT downregulated transcriptional activity of the promoter by 59 and 45 % in LNCaP and VCaP, respectively (*p* < 0.001 in both instances). The lower reduction seen in VCaP compared to LNCaP may reflect the presence of multiple androgen insensitive and constitutively active splice variants in the former cell line [39]. In contrast, DU145 cells, which lack AR, completely failed to respond to DHT (*p* > 0.85); however, cotransfection with the hAR expression plasmid pSVARo led to 48 % DHT-induced downregulation (*p* < 0.001). Treatment of DU145 cells expressing the hAR with the antiandrogens, bicalutamide (Bic) or enzalutamide (Enz) failed to repress luciferase activity (Fig. 4b, right panel). In addition, inclusion of antiandrogen with DHT antagonised the androgen-induced repression (data not shown). Therefore, the phAR1.6Luc plasmid behaved in a physiological manner and displayed agonist induced auto-downregulation that was mediated through the AR.

The next step was to mutate the putative ARE to create the reporter construct phAR1.6Luc-AREm (Fig. 4a) and determine the effect on transcriptional activity. In order to confirm TRANSFAC analysis that no new regulatory elements had been created by mutation, initial experiments were performed using PCa cells cultured in complete medium containing foetal bovine serum rather than charcoal stripped serum which is depleted in some components e.g. growth factors and hormones, to ensure that all cell signalling pathways were fully operational. Direct comparison of the WT hAR promoter and that containing the mutated 5′ UTR ARE in Fig 4c reveals that loss of the 5′ UTR ARE led to increases of 48 and 46 % in promoter activity in LNCaP and VCaP cells, respectively (both *p* < 0.001). While LNCaP cells express AR but not glucocorticoid receptor (GR), the converse occurs in DU145 cells [40], and in these cells, mutation of the 5′ UTR ARE had no effect (*p* > 0.34), thus confirming that no new regulatory element had been created, and that GR does not interact with this ARE. However, cotransfection of DU145 cells with the pSVARo hAR expression plasmid resulted in the 5′ UTR ARE mutation raising transcriptional activity by 25 % (Fig. 4c, *p* < 0.001). Together, these results show that AR binds to the 5′ UTR ARE to downregulate transcription and mutation of the site leads to a release of this repression.

Lastly, the role of the 5′ UTR ARE in contributing to androgen auto-downregulation of the hAR promoter was confirmed by looking at the effect of the site’s mutation on DHT-induced repression in LNCaP and VCaP cells. The results presented in Fig. 4d, in which DHT-induced repression is expressed as luciferase activity in 10 nM DHT relative to that in the absence of androgen, show that mutation of the 5′ UTR ARE diminished DHT repression from 59 to 43 % in LNCaP and from 45 to 24 % in VCaP (both *p* < 0.01). Interestingly, from Fig. 4d it can be seen that the luciferase reporter plasmids containing the mutated 5′ UTR ARE continue to be subject to androgen downregulation in both LNCaP and VCaP (*p* < 0.01 in both instances), albeit to a much lesser degree.

### hAR Binds to the Endogenous 5′ UTR ARE

Because the conditions of EMSA incubations cannot always reflect the chromatin environment, ChIP assays were undertaken to look at AR binding to the endogenous hAR 5′ UTR ARE site. In order to study the regulatory element in its native state and to compare the effects of its mutation, LNCaP cells were used directly or transiently transfected with either phAR1.6Luc or phAR1.6Luc-AREm containing the WT or mutated ARE, respectively, and treated with either vehicle or 10 nM DHT. Initial experiments to confirm the specificity of the PG21 anti-hAR antibody were performed using the well-characterised promoter and enhancer regions of the *psa* gene which contains three active AREs, and the results are shown in Supplemental Fig. 2. PCR amplification of the AREs demonstrated binding of the PG21 antibody; however, amplification of a region in the middle of the promoter distant from the AREs failed to produce a signal.

**Fig. 5:**
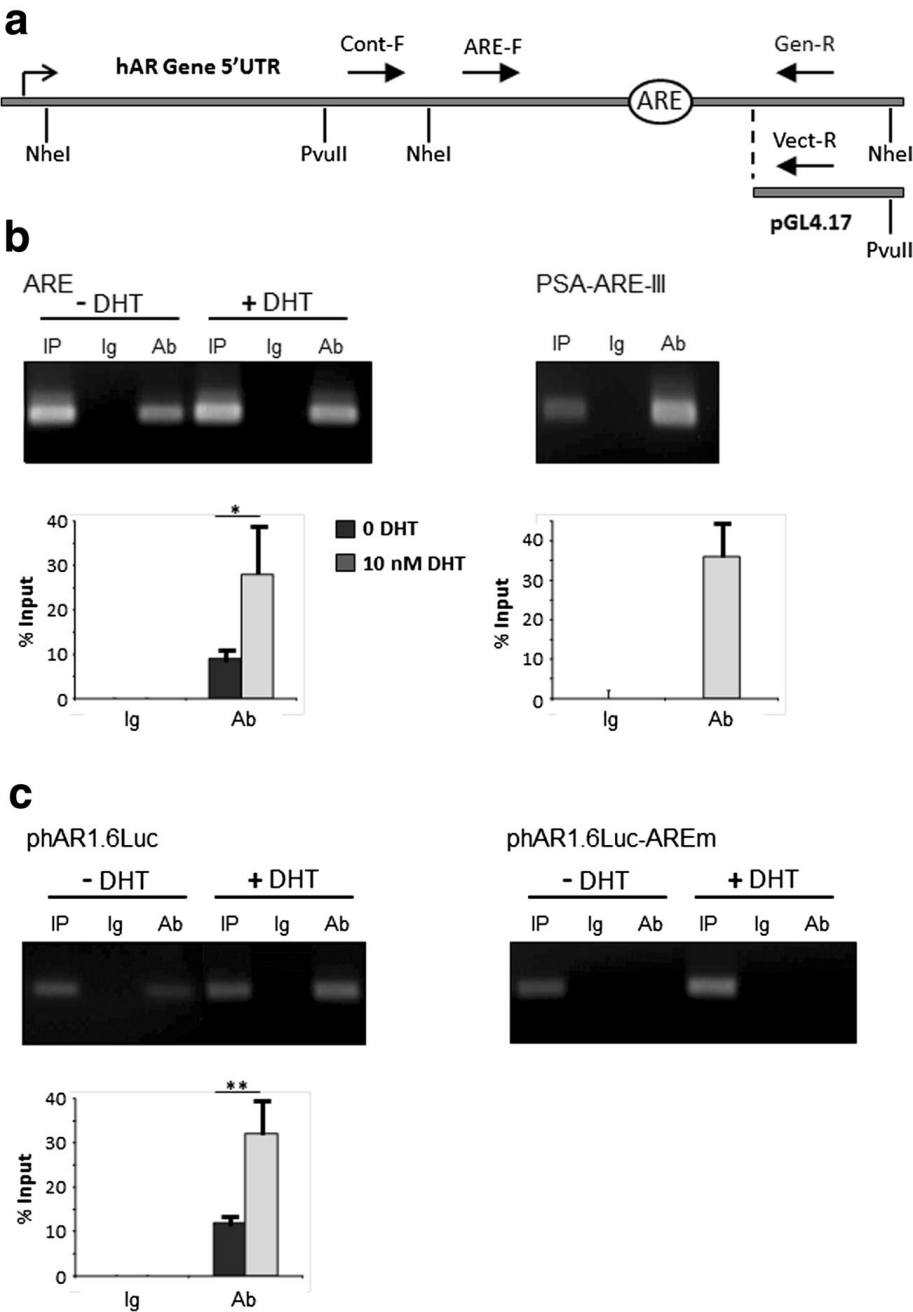
ChIP analysis confirms binding of *hAR* to 5′ UTR *ARE*. **a** *Line diagram* (not to scale) of the hAR 5′ UTR showing the recognition sites of the restriction endonucleases NheI and PvuII used to digest chromatin and plasmid, plus the forward (F) and reverse (R) primers (*solid arrows*) used for ChIP semiquantitative PCR. Oligonucleotides Gen-R and Vect-R are specific for the genomic and plasmid vector sequences, respectively, and the *bent arrow* indicates the transcriptional start site. **b**, **c** and **d** Representative agarose gels of PCR amplified immunoprecipitated DNA. **b** LNCaP cells were treated with either vehicle or 10 nM DHT (shown above gel) and ChIP was performed using PG21 anti-hAR antibody. Precipitated genomic DNA was amplified using primers for the ARE in the hAR 5′ UTR (*ARE*), or in the PSA upstream promoter (*PSA-ARE-III*) with DHT treated cells. *Lanes*: *IP* input sample, *Ig* preimmune rabbit IgG, *Ab* antibody. *Charts* display values expressed as percentage of input DNA and represent means ± S.D, *p < 0.05; **p < 0.01. **c** LNCaP cells were transfected with either phAR1.6Luc or phAR1.6Luc-AREm and subsequently treated with either vehicle or 10 nM DHT (both shown above gels). ChIP was carried out using PG21 anti-hAR antibody and the 5′ UTR ARE in precipitated plasmid amplified by PCR. *Lanes* and *charts* are as in panel **b**

Chromatin and plasmid were digested with NheI and PvuII in order to isolate the region of the 5′ UTR under study in the ChIP experiments from a previously described potential AR binding site proximal to the 5′ UTR ARE [30], and solubilised DNA was precipitated using anti-hAR antibody. Figure 5a depicts the hAR 5′ UTR along with the cleavage sites for NheI and PvuII, and the relative positions of the oligonucleotides used for semiquantitative PCR amplification. A forward primer upstream of the 5′ UTR ARE (ARE-F) was utilised in conjunction with either of two different reverse primers which were specific for the genomic sequence (Gen-R) or the plasmid vector (Vect-R). The effectiveness of endonuclease cleavage of both genomic chromatin and plasmid DNA was confirmed by PCR amplification of ChIP DNA input samples using the control forward oligonucleotide (Cont-F), which lies upstream of an NheI site (Fig. 5a), and the appropriate reverse primer (Supplemental Fig. 3).

Anti-hAR antibody, but not preimmune Ig, immunoprecipitated the 5′ UTR ARE region of the AR gene in LNCaP cells that had been treated with either vehicle or DHT (Fig. 5b). This result is in agreement with the fact that LNCaP cells express the T877A mutated form of AR which has a high constitutive transcriptional activity even in the absence of androgen [33]. Integration of unsaturated digital gel images and calculation of immunoprecipitated DNA relative to input DNA showed that treatment with 10 nM DHT led to a 2.5-fold increase in AR binding to the genomic 5′ UTR ARE (*p* < 0.05). PCR amplification of precipitated DNA with oligonucleotide primers encompassing the active ARE-III site in the human PSA upstream enhancer (Fig. 5b) confirmed the efficacy of the ChIP methodology. Similarly, anti-hAR antibody-precipitated phAR1.6Luc plasmid DNA in transfected LNCaP cells was PCR-amplified using the same 5′ UTR forward primer (ARE-F) and the vector-specific reverse primer (Vect-R). From Fig. 5c, it can be seen that, as with the genomic regulatory element, hAR binds to the 5′ UTR ARE in vivo both in the absence and presence of DHT. Similarly, integration of PCR gels showed that binding of AR to the ARE is increased 2.7-fold (*p* < 0.01) in the presence of androgen. On the other hand, hAR failed to bind to the 5′ UTR ARE of the luciferase reporter plasmid in which both half sites of the ARE had been mutated (phAR1.6Luc-AREm), regardless of the presence of hormone (*p* > 0.51 and *p* > 0.75 for vehicle and DHT, respectively).

## Discussion

Enduring expression of the androgen receptor in PCa contributes to tumour survival and proliferation as well as facilitating progression to fatal CRPC status. Therefore, it is vital to understand the molecular mechanics of human AR gene regulation; especially the negative feedback loop whereby ligand-activated AR downregulates transcription of its own gene, since androgen deprivation therapy (ADT) is the principal strategy of advanced PCa treatment regimes. In this study, we have identified an ARE in the 5′ UTR of the human AR gene and confirmed binding of AR to it by EMSA using both purified AR and LNCaP nuclear extract and ChIP assays. Importantly, luciferase measurements of transcriptional activity in PCa cell lines established that the 5′ UTR ARE downregulates expression in response to androgens, and disruption of this ARE alleviates repression in an AR-dependent manner. Furthermore, the clinically relevant antiandrogens, bicalutamide and enzalutamide were unable to mediate receptor-dependent repression. These observations were made independently of Vismara et al. [36] who suggested a putative nonclassical ARE in the 5′ UTR. In contrast to that study, we have demonstrated the binding of AR and functional relevance of this element in different prostate cell lines.

The adverse nature of the AR’s influence on PCa progression is manifested from the several lines of evidence. Androgen activation of AR limits proliferation of prostate epithelial cells in keeping with its role of promoting terminal differentiation [41, 42]. However, the AR usually increases proliferation of PCa cell lines [43, 44] and receptor inhibition results in repression of CRPC tumour growth [23]. Reduction of AR synthesis in both androgen sensitive (AS) and CRPC cells by RNA interference has revealed a correlation between AR expression and cell viability [45]. However, the escape of CRPC from hormone regulation is not due simply to increased amounts of AR, as the receptor directs expression of a distinct transcriptome that contributes to the ability to grow in an apparently androgen independent manner [46–48].

In order to maintain AR levels within narrow constraints in normal cells, the AR gene is under the control of multiple regulatory elements. In terms of hAR gene autoregulation, activated AR can stimulate expression through an exonic enhancer about 170 kbp distal from the promoter where specificity for AR is dependent upon the structure of the receptor’s NTD [31]. Androgen receptor auto-downregulation can occur through a repressor ARE in the second intron of the hAR gene 130 kbp downstream of the promoter [30] and the 5′ UTR ARE described in this report. Although sequence analysis reveals no other potential AREs in the promoter and 5′ UTR between −741 to +842 bp, the binding of AR to a site between −225 and +504 bp has been reported [30]. It must be emphasised that the repressor 5′ UTR ARE described in this report is distinct from that AR binding site as confirmed in the ChIP assays which differentiated between the two AR binding sites by restriction endonuclease digestion between them (Supplemental Fig. 3). The 5′ UTR ARE described here is also distinct from the AR binding site identified upstream of the gene promoter [49]. This site (chromosomal location 66,237–66,248 kbp) was associated with a binding site for the ETS transcription factor, ERG, and ERG-dependent repression of the AR gene.

Interestingly, while mutation of the 5′ UTR ARE in the reporter plasmid, which did not contain the intronic repressor ARE, significantly lessened AR-dependant downregulation in the PCa cell lines examined, this repression was not completely abolished (Fig. 4d). Our data are consistent with earlier observations that deletion of the section from +570 to +1,025 bp (containing the 5′ UTR ARE) in luciferase reporters driven by the hAR promoter and 5′ UTR does not completely abrogate downregulation by androgens [36]. A growing body of work has elucidated some of the signalling pathways by which this can occur through the regulatory elements present in the luciferase reporter (Fig. 1b). The dominant transcription factor driving AR expression is Sp1 that binds to several sites in the core promoter and 5′ UTR. DHT-activated AR can inhibit Sp1 transactivation without binding to chromatin by directly interacting with the transcription factor and interfering with its binding to its regulatory elements. Within PCa cells, this process has been found to downregulate Sp1-directed expression of c-Met in WR22Rv1 cells and LNCaP xenografts [50] and Smad3 in the PCa cell lines NRP-154AR, DU145AR, LNCaP and VCaP [51]. Another route operates through the transcription factor TWIST1 which upregulates hAR expression [52]. Expression of TWIST1 is repressed by androgens in PCa cells through a process mediated by NKX3-1. In brief, androgens strongly upregulate NKX3-1 production in prostate epithelial cells [53, 54] and in PCa cells where upon NKX3-1 binds to the TWIST1 promoter to strongly repress transcription [55]. An active cyclic AMP-responsive element (CRE) in the hAR promoter [56] increases AR transcription in response to cAMP signalling. Members of the CREB/ATF family bind to CRE sites and in turn bind CREB binding protein (CBP) which forms a bridge with the basal transcription apparatus. Androgen-activated AR in PCa cells can sequester CBP without binding to DNA, thereby squelching transcription involving CBP [57]. Another member of the CREB/ATF family that binds CRE sites, ATF3, is a repressor [58] and is overexpressed in many cancer cells. In LNCaP, its expression is strongly upregulated by DHT [54] which would lead to downregulation of promoters with CRE sites. Lastly, two of the eight NF-κB binding sites in the hAR promoter proximal to the initiation site that increase AR transcription upon binding of p 50 and RelA (p65) in PCa cells [59] are present in the reporter plasmid. Androgen activation of AR reduces expression, nuclear localisation and transcriptional activity of RelA in PCa cells [60]. Together, auto-repression of the hAR gene through multiple avenues provides redundancy to protect against the consequences of mutation and a means of fine tuning expression of a powerful developmental gene.

One of the routes by which AR regulates target gene expression is through epigenetic remodelling of chromatin. An example is the recruitment of lysine-specific demethylase 1 (LSD1) by activated AR to its associated ARE where it can behave as a corepressor or coactivator [61]. Indeed, the aforementioned ARE repressor regulatory element in the second intron has recently been shown to downregulate AR expression through AR binding and the action of LSD1 [30]. However, we found no evidence that LSD1 is recruited to the ARE in the 5′ UTR (Supplemental Fig. 4); therefore, another of the many mechanisms of ARE-based repression is most likely involved [62].

Another significant finding was that the 5′ UTR ARE is confined to primates with the equivalent region in other species, especially the rodents, rat and mouse, displaying particularly low homology. This strongly suggests that caution should be exercised when using rodent models to investigate regulation of the AR gene.

In conclusion, pertinacious AR signalling is implicated in the progression to CRPC with the levels of the receptor mediating the life and death of tumours. The repressor ARE we have characterised in the 5′ UTR lends valuable insight into the control of AR expression and will provide targets for novel therapeutic agents against CRPC. The finding that AR expression is downregulated by androgens through multiple sites raises the question of whether ADT can on occasion be counterproductive as a consequence of transcriptional repression being alleviated. In addition, DHT can have an indirect protective role as it has recently been found to inhibit the induction of autoimmune and inflammatory responses in human prostatic stromal cells [63]. Thus, the benefits of ADT must be balanced with a consideration of the risks and perhaps more attention should be focused on bipolar androgen therapy (BAT) [64, 65] in which acute ablation and supraphysiologic levels of androgen are alternated in rapid cycles to prevent PCa cells adapting their AR expression in response to environmental conditions.

## Electronic Supplementary Material

Below is the link to the electronic supplementary material.

Supplemental Fig. 1 (GIF 125 kb)

High resolution image (TIFF 122 kb)

Supplemental Fig. 2 (GIF 112 kb)

High resolution image (TIFF 105 kb)

Supplemental Fig. 3 (GIF 75 kb)

High resolution image (TIFF 29 kb)

Supplemental Fig. 4 (GIF 72 kb)

High resolution image (TIFF 20 kb)

Supplementary Methods and Figure legends (DOCX 37 kb)
