# Supplementary figures and images for: Negative Regulation of the Androgen Receptor Gene Through a Primate-Specific Androgen Response Element Present in the 5′ UTR
Source: Horm Cancer. 2014 Jun 4;5(5):299–311. doi: 10.1007/s12672-014-0185-y (PMC4164857; doi:10.1007/s12672-014-0185-y)

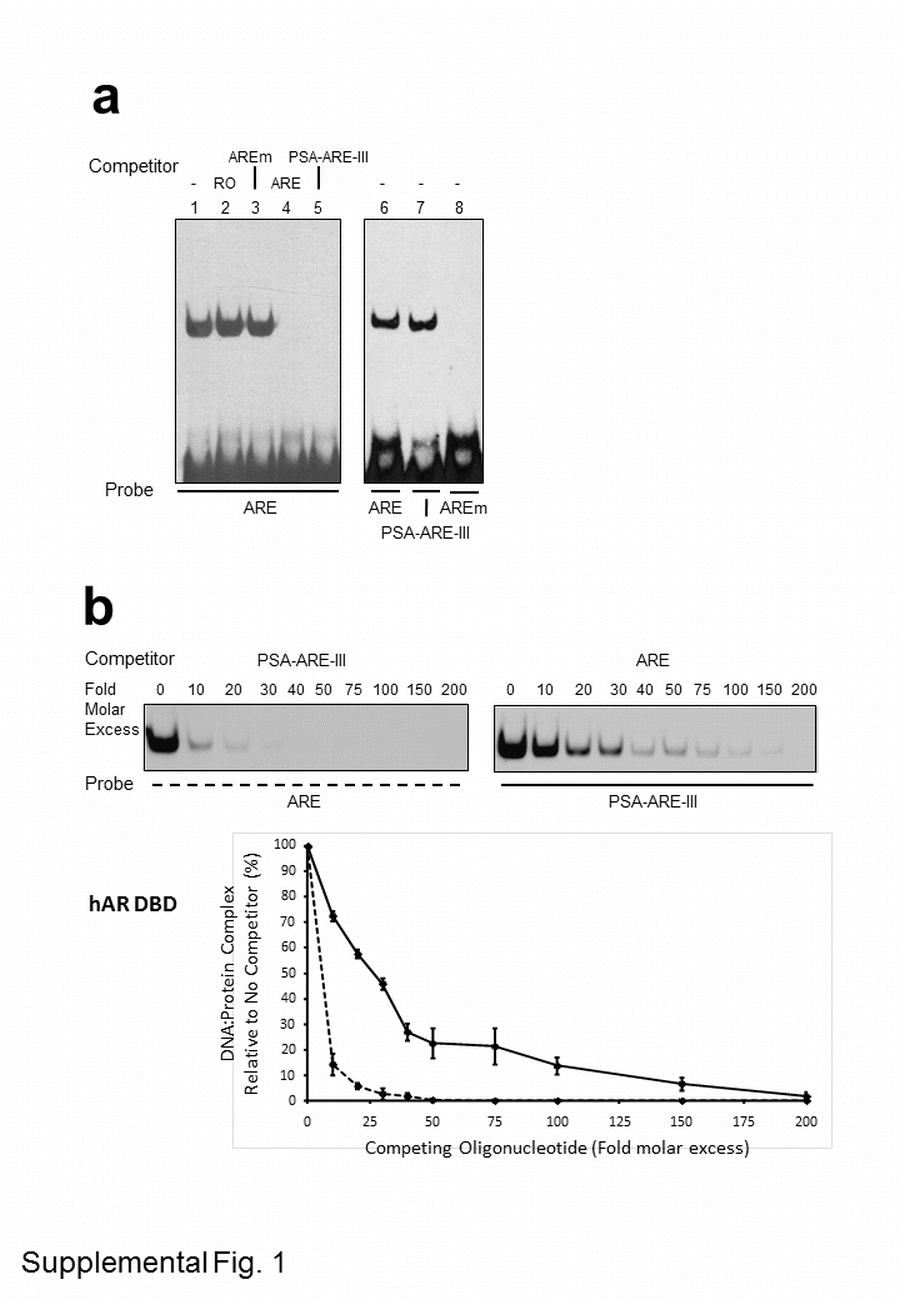

Supplement: Supplementary file 1 — (GIF 125 kb) [file 12672_2014_185_Fig6_ESM.gif]

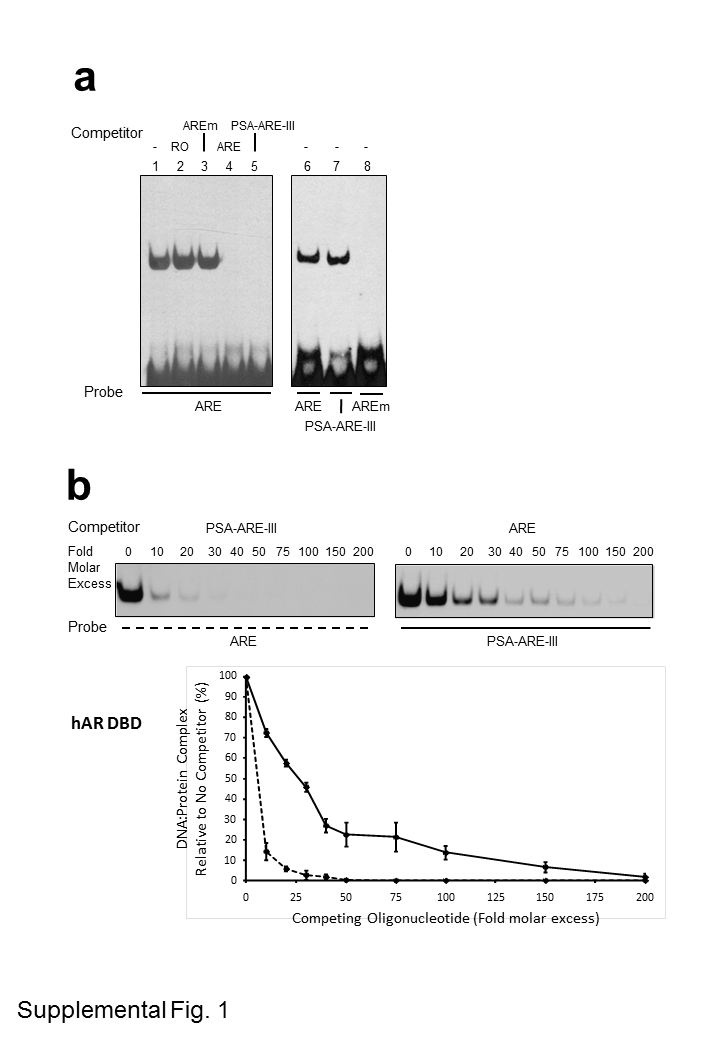

Supplement: Supplementary file 2 — High resolution image (TIFF 122 kb) [file 12672_2014_185_MOESM1_ESM.tif]

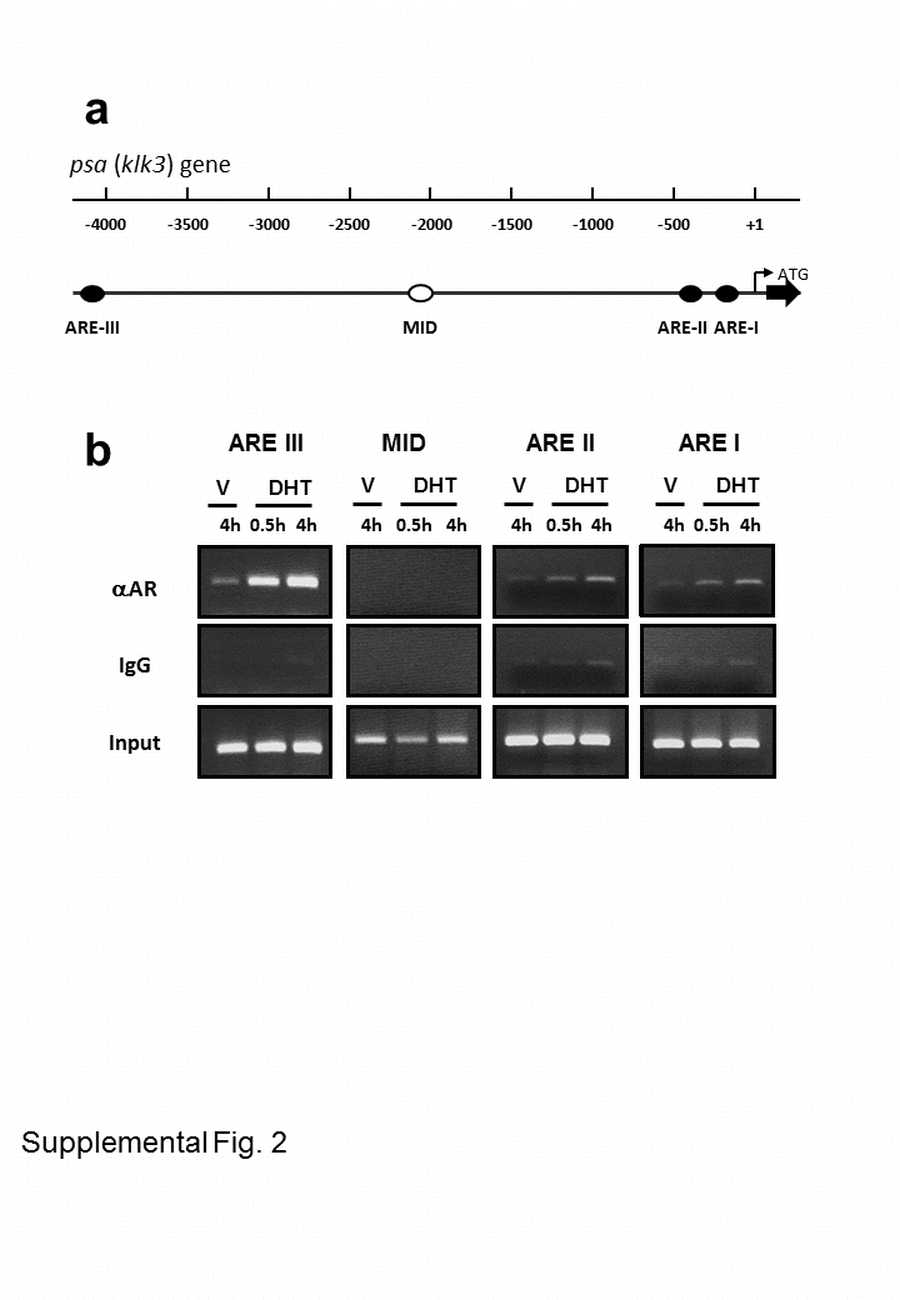

Supplement: Supplementary file 3 — (GIF 112 kb) [file 12672_2014_185_Fig7_ESM.gif]

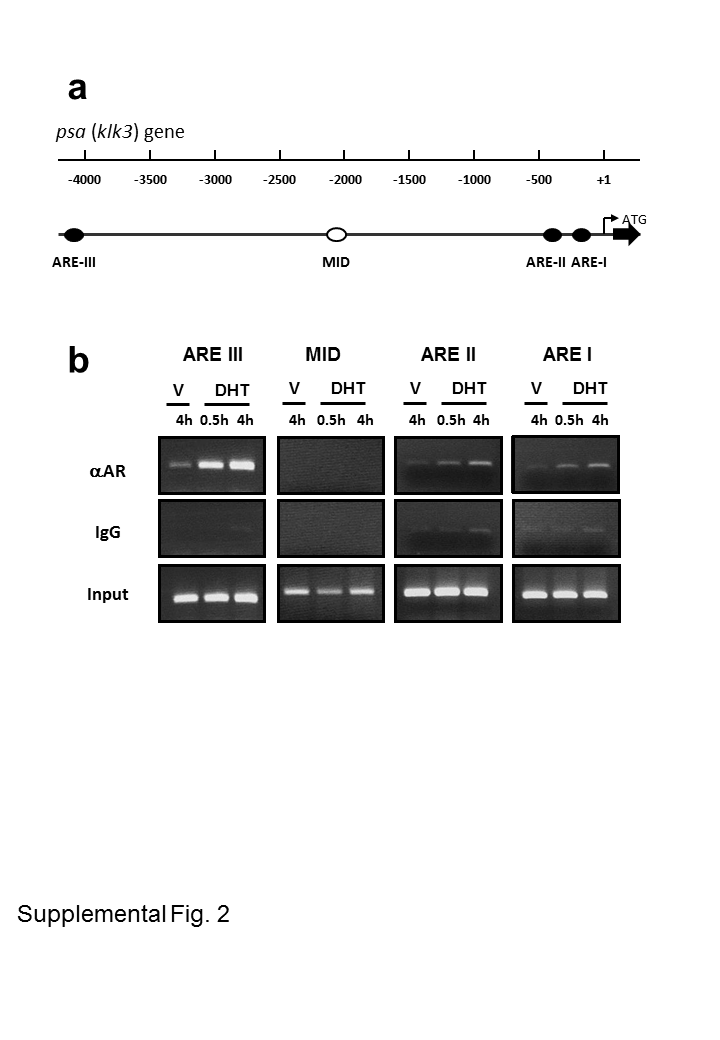

Supplement: Supplementary file 4 — High resolution image (TIFF 105 kb) [file 12672_2014_185_MOESM2_ESM.tif]

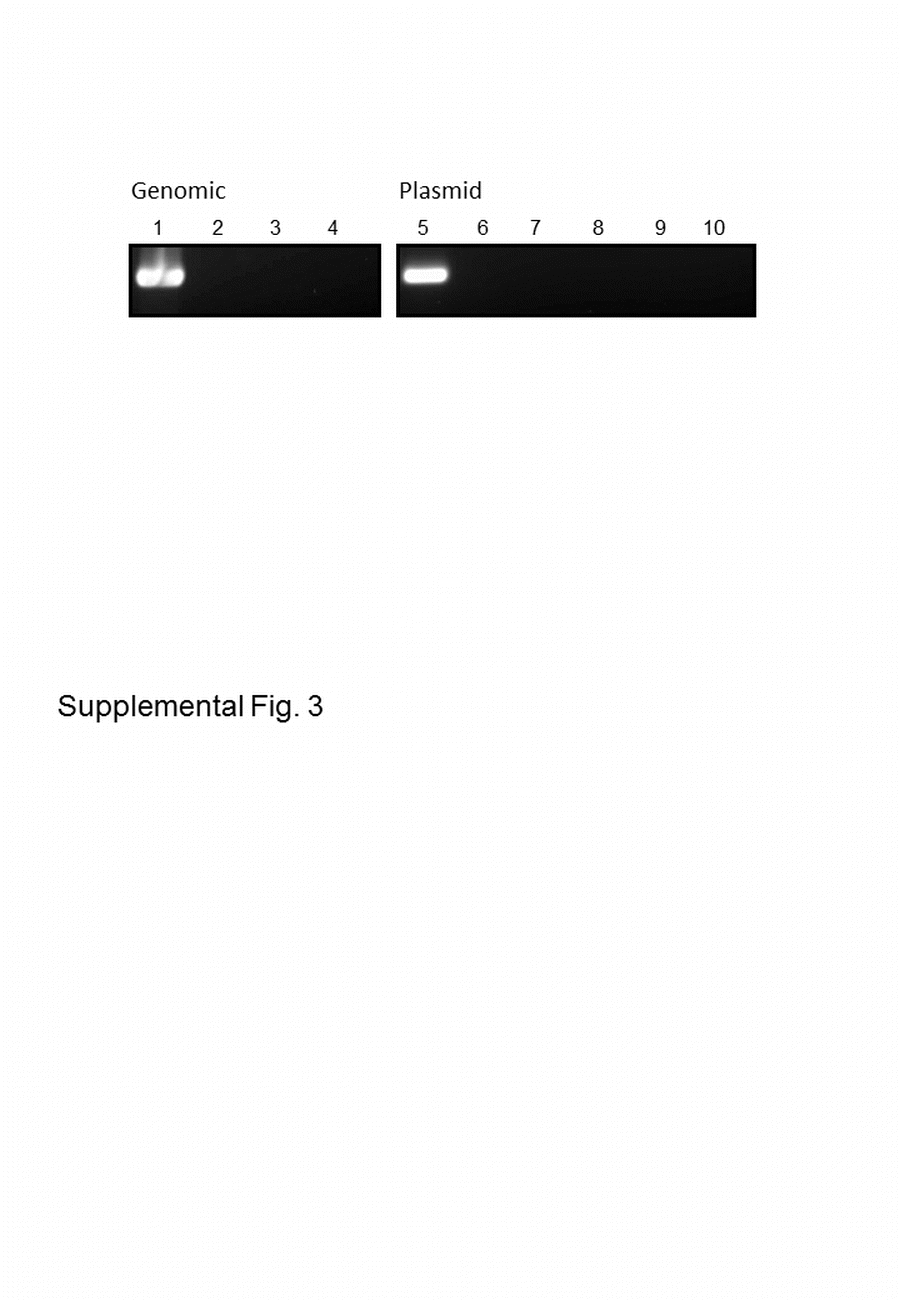

Supplement: Supplementary file 5 — (GIF 75 kb) [file 12672_2014_185_Fig8_ESM.gif]

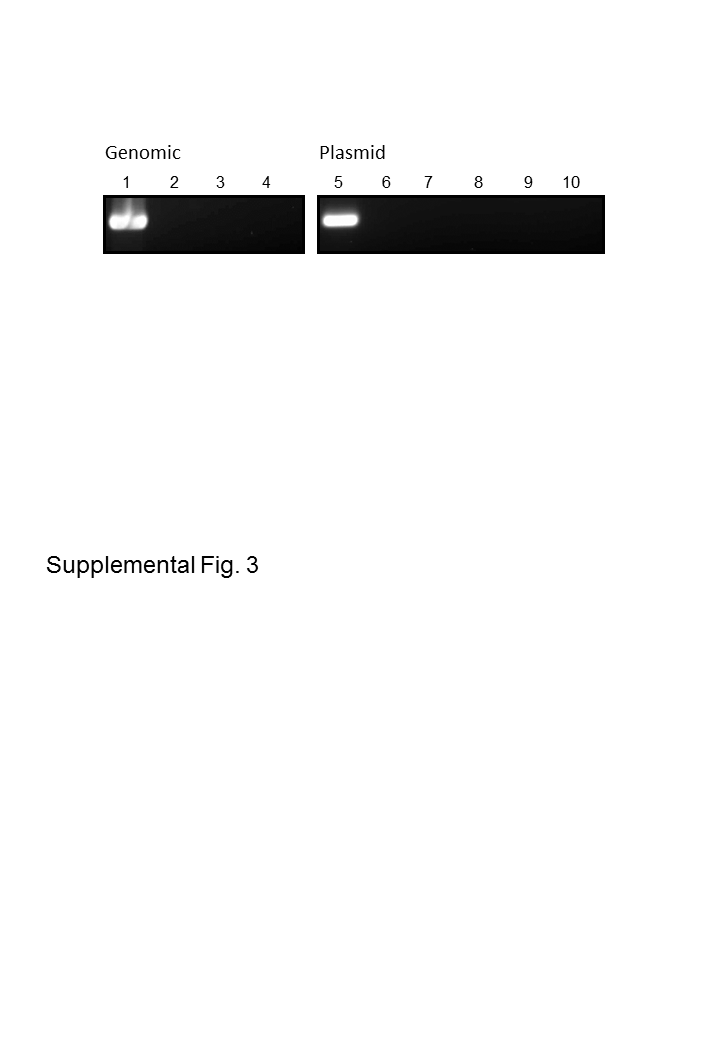

Supplement: Supplementary file 6 — High resolution image (TIFF 29 kb) [file 12672_2014_185_MOESM3_ESM.tif]

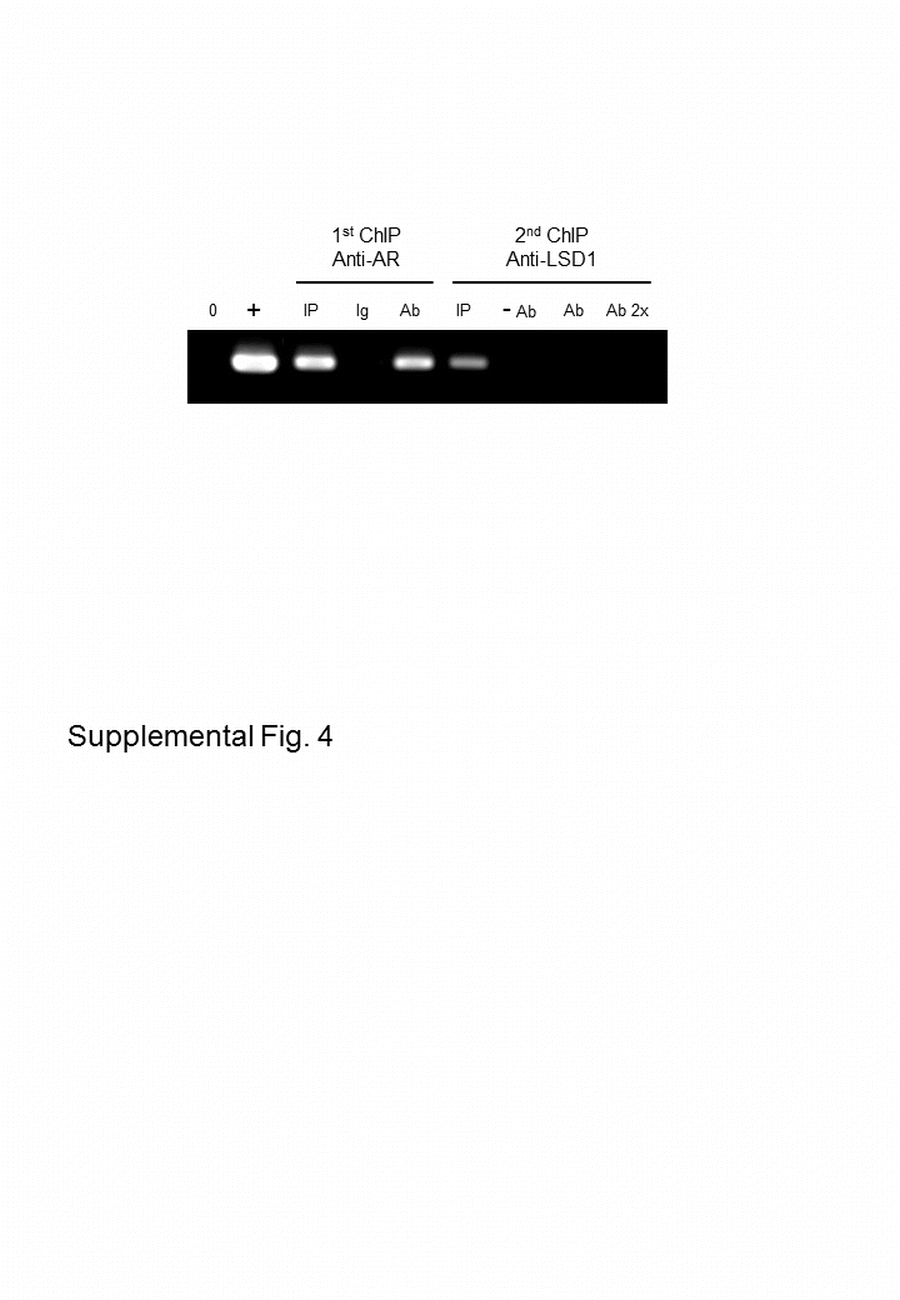

Supplement: Supplementary file 7 — (GIF 72 kb) [file 12672_2014_185_Fig9_ESM.gif]

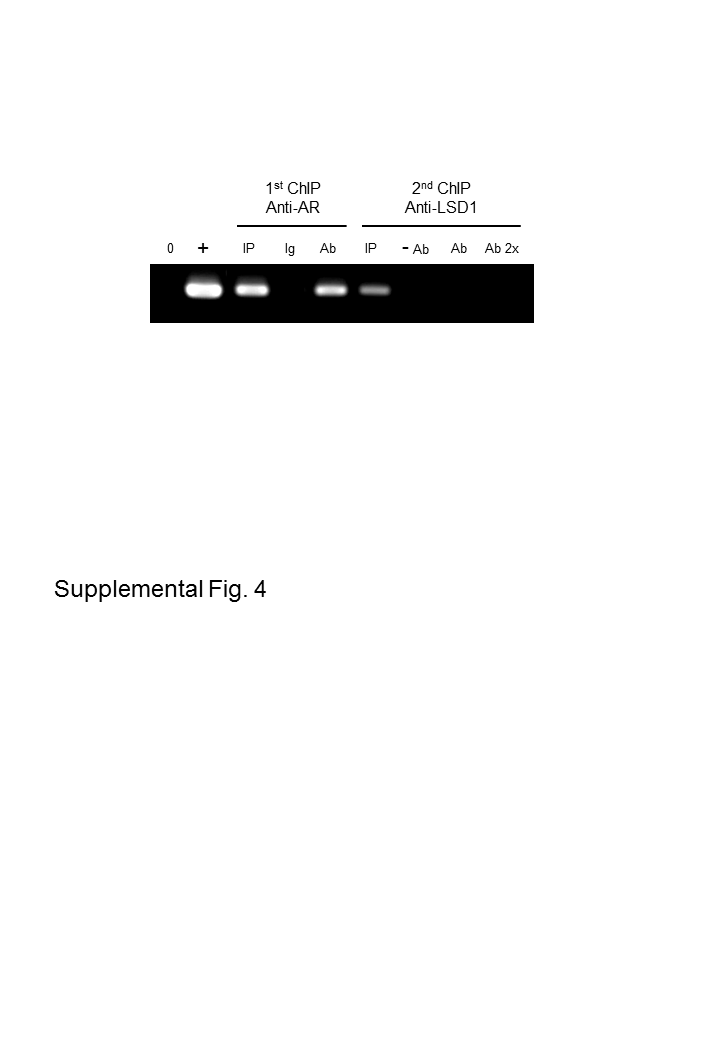

Supplement: Supplementary file 8 — High resolution image (TIFF 20 kb) [file 12672_2014_185_MOESM4_ESM.tif]
