# Supplementary material for: Negative Regulation of the Androgen Receptor Gene Through a Primate-Specific Androgen Response Element Present in the 5′ UTR
Source: Horm Cancer. 2014 Jun 4;5(5):299–311. doi: 10.1007/s12672-014-0185-y (PMC4164857; doi:10.1007/s12672-014-0185-y)
Supplement: Supplementary file 9 — Supplementary Methods and Figure legends (DOCX 37 kb) [file 12672_2014_185_MOESM5_ESM.docx]

**Supplementary Material**

**Supplemental Methods**

Chromatin immunoprecipitation (ChIP) assay

ChIP assays were performed with the addition of protease inhibitors (complete protease inhibitor cocktail from Roche and 1.0 mM PMSF) and protein phosphatase inhibitors (5 mM β-glycerophosphate and 100 µM Na_3_VO_4_). LNCaP cells were transfected with either phAR1.6Luc or phAR1.6Luc-AREm, encoding the wild type and mutated ARE respectively, and grown to approximately 90% confluency on 15 cm tissue culture plates in complete medium supplemented with 10% charcoal stripped serum (PAA). The medium was replaced with fresh charcoal-stripped medium containing either 10 nM DHT (Sigma) or ethanol vehicle and incubated for 4 h. The media were aspirated and replaced with phosphate buffered saline (PBS) containing 1% formaldehyde followed by incubation at 37°C for 10 min. Glycine was added to a final concentration of 125 mM and the plates were incubated at room temperature for 5 min with gentle rocking, after which the glycine solution was removed and the cells washed twice with cold PBS.

Prepared nuclei were sonicated for preliminary optimisation experiments or resuspended in cold restriction endonuclease buffer (180 μl/Petri dish) for subsequent investigation of the 5’UTR ARE. In the latter case, chromatin and plasmid were digested with 400 units each PvuII (NEB) and NheI (Roche) for 15 min at 37˚C. The reaction was stopped with SDS lysis buffer to give a final volume of 680 µl containing 1% SDS, 10 mM EDTA and 50 mM Tris pH8.1, then vortexed briefly and incubated on ice for 15 min. Following centrifugation at 13000 x g for 10 min at 4˚C, the supernatant was made up to 6 ml with ChIP dilution buffer containing 1 µg/ml sonicated salmon sperm DNA and 10 mg/ml BSA. The lysate was precleared by rotation for 30 min at 4˚C using 100 µl each Protein‑G and Protein‑A Dynabeads (Life Technologies) which had been prepared as per manufacturer's instructions and with addition of 1 mg/ml BSA and 1 μg/ml sheared salmon sperm DNA. Lysate was divided into three aliquots and the beads removed by magnetisation. The cleared lysates were transferred to fresh tubes and either 5 µg anti-hAR antibody (PG21, 06-680 Millipore) or IgG were added to individual samples, which were subsequently incubated overnight at 4˚C with gentle rotation. Samples of cleared lysates were retained as input (IP). Immunoprecipitations were isolated by adding 30 µl each Protein‑G and Protein‑A Dynabeads per sample followed by rotation for 2 h at 4˚C. The beads were collected and washed twice each with low salt buffer, high salt buffer, LiCl wash and TE solution. Similarly, reversal of crosslinking, and removal of RNA and proteins were done as before. DNA was purified using MinElute Spin Columns (Qiagen) which were eluted twice with molecular biology grade water.

Re-ChIP was performed sequentially using two 15 cm Petri dishes of untransfected LNCaP cells which were cultured and treated with 10 nM DHT as above. The first round of ChIP was carried out as normal using anti-hAR antibody PG21 and immunocomplexes were eluted from the washed Dynabeads by incubation in 10 mM DTT for 30 min at 37˚C. A fifty fold volume of dilution buffer (1% Triton X‑100, 150 mM NaCl, 2 mM EDTA and 20 mM Tris pH8.1) was added and the second round of ChIP was carried out with anti-LSD1 antibody (Millipore 05-939) in a similar manner to the single ChIP assays.

Semi-quantitive PCR was performed using 1 - 2 µl DNA and 24 cycles or 2 - 4 µl DNA and 42 cycles to amplify plasmid and genomic templates respectively, using HotStarTaq DNA Polymerase (Qiagen). The forward (F) and reverse (R) primers were: ARE‑F, 5’‑CATTGCAAAGAAGGCTCTTAGG‑3’; Cont‑F, 5’-CCCGAGTTTGCAGAGAGGTA-3’; Gen‑R, 5’‑GGACAAGATCTGCCCTGCTA‑3’; Vect‑R, 5’‑TCTTCCATGGTGGCTTTACC‑3’; PSA‑ARE‑III‑F, 5’‑GGTGAGAAACCTGAGATTAGGAATC‑3’; PSA‑ARE‑III‑R, 5’‑GTGTGTCTTCTGAGCAAAGACAGC‑3’; PSA‑MID‑F 5’‑ctcactggagtggagacaaacttc‑3’; PSA‑MID‑R 5’‑cctggcaaacattggtgaaacccc‑3’; PSA‑ARE‑II‑F 5’‑GCTAGGGAACTTTGGGAGACTC‑3’; PSA‑ARE‑II‑r 5’‑CGATTCTTCATACAAAGCCTCACG‑3’; PSA‑ARE‑I‑f 5’‑CCTGAGTGCTGGTGTCTTAGGGC‑3’and PSA‑ARE‑I‑R 5’‑GGAGCCCTATAAAACCTTCATTCCCC‑3’ . The resulting DNA products were resolved by 2.0% agarose gel electrophoresis in TAE buffer and visualised by ethidium staining.

**Supplemental Figures**

**Supplemental Fig. 1** Additional studies of hAR binding to the 5’UTR ARE. Purified hAR protein encoding the DBD (residues 529–645) was incubated with the labelled oligonucleotide probes indicated below each gel and the products resolved by electrophoretic mobility shift analysis. **a** The 5’UTR ARE (ARE) and PSA‑ARE‑III both bind hAR DBD but the mutated AREM does not. Competing unlabelled oligonucleotides (100 fold molar excess) were added prior to addition of probe and are shown above the gels; RO, a random oligonucleotide. EMSAs are representative of at least 3 independent experiments. **b** The non-consensus 5’UTR ARE has lower affinity for hAR DBD than does a consensus ARE. Human AR DBD was incubated with either labelled ARE probe and competed by preincubation with PSA‑ARE‑III oligonucleotide (*dashed line*) or labelled PSA‑ARE‑III probe and competed with ARE oligonucleotide (*solid line*). The molar excess of unlabelled competing oligonucleotide is shown above representative EMSAs. Data presented in the graphs were generated using unsaturated images, and the values are the means of a minimum of three independent experiments ± SD.

**Supplemental Fig. 2** ChIP analysis confirms specificity of anti-hAR antibody. **a** Line diagram of the *psa (klk3)* promoter showing the three AREs (*solid symbols*) and the middle region devoid of AR binding sites (*open symbol*) that is amplified by the MID PCR primers. Bent arrow indicates the transcriptional start site (+1) and ATG with solid arrow show the start of translation. **b** Aagarose gels of PCR amplified immunoprecipitated DNA. LNCaP cells were treated with either vehicle (V) or 10 nM DHT for 0.5 h and 4 h (*shown above gel*) and ChIP was performed using PG21 anti-hAR antibody. IP, input sample.

**Supplemental Fig. 3** PCR amplification of ChIP input samples using forward control primer (Cont‑F) and reverse primer specific for genomic or plasmid template; Gen‑R and Vect‑R respectively. Lane 1, male human genomic DNA (PCR positive control); lane 2, male human genomic DNA digested with NheI and PvuII; lanes 3 and 4, ChIP input from LNCaP cells treated with vehicle or 10 nM DHT respectively; lane 5, phAR1.6Luc DNA; lane 6, phAR1.6Luc DNA digested with NheI and PvuII; lanes 7 and 8, ChIP input from LNCaP cells transfected with phAR1.6Luc and treated with vehicle or 10 nM DHT respectively; lanes 9 and 10, as lanes 7 and 8 except that cells were transfected with phAR1.6Luc-AREm.

**Supplemental Fig. 4** Re-ChIP of LNCaP cells with anti‑LSD1. Two rounds of ChIP were carried out on DHT treated LNCaP cells with the first round being performed as for Fig. 5, panel b using anti-hAR antibody and the second round with anti-LSD1 antibody. Lanes: 0, no DNA template; +, PCR positive control using male human genomic DNA; IP, input sample; Ig, pre-immune rabbit IgG; Ab, antibody; and Ab 2x, double the amount of anti-AR precipitated chromatin starting material for the anti-LSD1 ChIP.
